# Stereotactic Ablative Radiosurgery for Locally Advanced or Recurrent Skull Base Malignancies with Prior External Beam Radiation Therapy

**DOI:** 10.3389/fonc.2015.00065

**Published:** 2015-03-17

**Authors:** Karen M. Xu, Kimmen Quan, David A. Clump, Robert L. Ferris, Dwight E. Heron

**Affiliations:** ^1^Department of Radiation Oncology, University of Pittsburgh Cancer Institute, Pittsburgh, PA, USA; ^2^Department of Otolaryngology-Head and Neck Surgery, University of Pittsburgh Cancer Institute, Pittsburgh, PA, USA

**Keywords:** SABR, low toxicities, re-irradiation, skull base malignancies, high-dose

## Abstract

**Purpose:** Stereotactic ablative radiotherapy (SABR) is an attractive modality to treat malignancies invading the skull base as it can deliver a highly conformal dose with minimal toxicity. However, variation exists in the prescribed dose and fractionation. The purpose of our study is to examine the local control, survival, and toxicities in SABR for the treatment of previously irradiated malignant skull base tumors.

**Materials and methods:** A total of 31 patients and 40 locally advanced or recurrent head and neck malignancies involving the skull base treated with a common SABR regimen, which delivers a radiation dose of 44 Gy in 5 fractions from January 1st, 2004 to December 31st, 2013, were retrospectively reviewed. The local control rate (LC), progression-free survival rate, overall survival (OS) rate, and toxicities were reported.

**Results:** The median follow-up time of all patients was 11.4 months (range: 0.6–67.2 months). The median tumor volume was 27 cm^3^ (range: 2.4–205 cm^3^). All patients received prior external beam radiation therapy with a median radiation dose of 64 Gy (range: 24–75.6 Gy) delivered in 12–42 fractions. Twenty patients had surgeries prior to SABR. Nineteen patients received chemotherapy. Specifically, eight patients received concurrent cetuximab (Erbitux™) with SABR. The median time-to-progression (TTP) was 3.3 months (range: 0–16.9 months). For the 29 patients (93.5%) who died, the median time from the end of first SABR to death was 10.3 months (range: 0.5–41.4 months). The estimated 1-year OS rate was 35%. The estimated 2-year OS rate was 12%. Treatment was well-tolerated without grade 4 or 5 treatment-related toxicities.

**Conclusion:** Stereotactic ablative radiotherapy has been shown to achieve low toxicities in locally advanced or recurrent, previously irradiated head and neck malignancies invading the skull base.

## Introduction

Skull base tumors (SBT) may originate from various tissues of the skull base or from direct extensions of head and neck cancers (1). The skull base is also a common site of metastasis from distant tumors (2, 3). Common clinical presentations include pain and cranial nerve deficits, such as visual disturbances, facial paresis, dysphagia, and odynophagia, which bring great suffering to the patients (4). However, due to their close proximity to critical neurovascular structures, treatment of malignant tumors involving the skull base presents a difficult challenge to the clinician, especially when such tumors persist or recur after surgery and/or external beam radiation therapy (EBRT) (5).

Recently, fractionated stereotactic radiotherapy has become an attractive modality to re-irradiate skull base malignancies since it can deliver a highly conformal dose to the tumor while minimizing radiation to surrounding critical structures (6–9). However, there is no consensus on the stereotactic dose and fractionation. In this study, we report our institution’s experience using linear accelerator-based stereotactic ablative radiotherapy (SABR) for the treatment of locally advanced or recurrent skull base malignancies.

## Materials and Methods

### Patient population

With approval from our institutional review board (IRB), we performed a retrospective review of 31 patients with 40 locally advanced or recurrent, previously irradiated skull base malignancies treated with high-dose fractionated SABR at our institution from January 1, 2004 to December 31, 2013 with Eastern Cooperative Oncology Group (ECOG) performance status of 0–2.

### Simulation and planning

Each patient received pretreatment skull based MRI or ^18^F-fluorodeoxy-glucose (^18^F-FDG) PET/CT scans, which were fused with contiguous ≤2.5-mm-thick slice CT treatment planning images using commercially available fusion software. Our methods for the use of ^8^F-FDG PET/CT scans in head and neck cancers were described previously (10). Patients were placed in a supine position in an alpha cradle both during CT imaging and the treatment and immobilized with a rigid thermoplastic Aquaplast™ facemask (WRF/Aquaplast Corp., Wyckoff, NJ, USA). The tumor volume and surrounding critical structures were contoured by a radiation oncologist and a head and neck surgeon. Quality assurance testing of the treatment plan was based on phantom dose measurements by a radiation physicist. An ideal SABR treatment plan provided coverage of 95% of the prescription dose to the PTV while sparing surrounding critical organs such as the left and right eye, left and right optic nerve, chiasm, brainstem, and spinal cord.

### Stereotactic radiotherapy delivery systems

Three platforms were used: Cyberknife™, Varian Trilogy™, and Truebeam™ STX (11). Cyberknife™ uses a compact 6-MV linear accelerator mounted on a computer-controlled robotic arm with six rotation axes that permit the use of 1200 treatment positions, of which 80–120 are usually necessary to treat most lesions. Throughout the treatment delivery, two orthogonally positioned diagnostic x-ray cameras provide images of the patient’s anatomy. Bony landmarks or implanted fiducial markers were used to compare the patient’s planning CT to allow for continuous adjustment (intra-fraction correction) based on the patient’s positioning (12). For Varian Trilogy™ and Truebeam™ STX, a cone-beam CT was acquired and pre-treatment shifts were made to match the planning scan after immobilization of the patient and isocentric set-up. Via beam modulation and occasionally using RapidArc™ technology, dose is delivered both efficiently and conformally (13, 14). For the 40 locally advanced or recurrent malignant skull base tumors (SBT) in our study, 26 were treated with Cyberknife™, 8 were treated with Varian Trilogy™, and 6 were treated with Truebeam™ STX.

### Clinical assessment and follow-up

Follow-up typically began 1 month after the completion of SABR. Patients were subsequently followed in 3- to 4-month intervals afterwards. During each follow-up visit, a clinical evaluation and physical examination were performed. MRI or PET-CT imaging studies were also obtained to assess any changes in tumor size or to identify the development of any new lesions. The follow-up duration was calculated from the end of SABR to the most recent follow-up time or in most cases, the cease to breathe date.

### Data analysis

Tumor response to the treatment was graded using the Response Evaluation Criteria in Solid Tumors (RECIST) criteria. Local failure (LF) was defined as any progression of disease in the target volume of the SABR. Regional failure (RF) was defined as any progression of disease in regional lymph nodes. Distant failure (DF) was defined as any progression of disease outside the target volume of the SABR, and not RF. Progression-free survival (PFS) was defined as any progression (local, regional, or distant) from the completion date of SABR. Overall survival (OS) defined as the time from the completion of the first SABR to the date of death. Survival curves and median survival time were estimated using the Kaplan–Meier method. All statistical tests were run using SPSS Version 22.0 (SPSS, Chicago, IL, USA) with a *p* value <0.05 considered statistically significant. Acute (<90 days) and late (>90 days) toxicities were assessed at follow-up visits approximately every 3 months after the treatment was complete. At each visit, toxicities were recorded based on the National Cancer Institute (NCI) Common Terminology Criteria for Adverse Events (CTCAE) Version 4.0. For this study, we gathered toxicity data retrospectively through patient chart review.

## Results

### Patient characteristics

Between January 2004 and December 2013, 31 patients with 40 locally advanced or recurrent, previously irradiated skull base malignancies were treated with SABR. The median age of the patients was 58.6 years old (range: 32.3–87.4 years old). Eighteen patients were males and 13 were females. The median follow-up time of all patients was 11.4 months (range: 0.6–67.2 months). Except two patients, all (*n* = 29) had a follow-up duration of more than 90 days. The median tumor volume was 27 cm^3^ (range: 2.4–205 cm^3^). Primary locations of tumors included oropharynx, nasopharynx, maxillary sinus, parotid gland, base of skull, salivary gland, tonsil, thyroid, retromolar trigone, ear canal, paranasal sinus, base of tongue, adenoid, and head and neck. Histology of tumors included squamous cell carcinoma, adenoid cystic carcinoma, adenocarcinoma, olfactory neuroblastoma, small cell carcinoma, medullary carcinoma, malignant fibrous histiocytoma, and the undifferentiated. The results were summarized in Table 1.

**Table 1:** **Patient characteristics**.

Characteristics	No. (%)
**Age (years)**
Median	58.6
Range	32.3–87.4
**Gender**
Male	18 (58)
Female	13 (42)
**Follow-up (months)**
Median	11.4
Range	0.6–67.2
**Tumor volume (cc)**
Median	27
Range	2.4–205
**Primary sites**
Oropharynx	3 (9.7)
Nasopharynx	7 (22.6)
Maxillary sinus	3 (9.7)
Parotid gland	3 (9.7)
Base of skull	3 (9.7)
Salivary gland	1 (3.2)
Tonsil	3 (9.7)
Thyroid	1 (3.2)
Retromolar trigone	1 (3.2)
Ear canal	1 (3.2)
Paranasal sinus	1 (3.2)
Base of tongue	2 (6.5)
Adenoid	1 (3.2)
Head and neck	1 (3.2)
**Histology**	
Squamous cell carcinoma	17 (54.8)
Adenoid cystic carcinoma	6 (19.4)
Adenocarcinoma	1 (3.2)
Olfactory neuroblastoma	1 (3.2)
Small cell carcinoma	1 (3.2)
Medullary carcinoma	1 (3.2)
Malignant fibrous histiocytoma	1 (3.2)
Undifferentiated	2 (6.5)
Unknown	1 (3.2)

### Treatment regimen

All patients received prior EBRT with a median radiation dose of 64 Gy (range: 24–75.6 Gy) delivered in 12–42 fractions. Twenty patients received prior surgery. Nineteen patients received chemotherapy, either chemotherapy prior to SABR or concurrent chemoradiation. Specifically, eight patients received concurrent cetuximab (Erbitux™) with SABR. The biologically effective dose (assuming an alpha/beta ratio of 10, for acute responding tissues or tumor effects), BED_10_, received by patients before SABR, was calculated for each patient according to the formula BED_10_ (Gy) = total dose × [1 + (Dose per fraction)/10]. The median BED_10_ was 82.7 Gy (range: 22.5–100 Gy). The biologically effective dose (assuming an alpha/beta ratio of 3, for late responding tissues or normal organ effects), BED_3_, received by patients before SABR, was calculated for each patient according to the formula BED_3_ (Gy) = Total dose × [1 + (dose per fraction)/3]. The median BED_3_ was 173.1 Gy (range: 40–216.7 Gy). The homogeneity index (HI) was calculated for each treatment plan. The HI describes the uniformity of dose within a treated target volume and is calculated according to the formula HI = maximum dose/prescription dose. The median HI was 1.3 (range: 1.1–1.3). The median SABR dose was 44 Gy (range: 15–50 Gy) and was delivered at a median isodose line of 80% (range: 75–94%) in one to five fractions. The median treatment duration was 10.5 days (range: 1–34 days). All patients completed the treatment course without toxicity-related breaks.

### Treatment response, tumor control, and survival

Out of the 40 locally advanced or recurrent skull base malignant tumors treated with SABR, 3 (7.5%) had complete response (CR); 7 (17.5%) had partial response (PR); 12 (30%) had stable disease (SD); and 9 (22.5%) had progressive disease (PD). The treatment responses for nine (22.5%) tumors were unknown mostly because the post-treatment imaging was unavailable.

The median follow-up time for all 31 patients was 11.4 months (range: 0.6–67.2 months). At the most recent follow-up, or at the time of death, 20 out of 40 (50%) SABR treatments had LF only. Six treatments (15%) had both local and DF; one (2.5%) treatment had both regional and DF; three (7.5%) treatments had local, regional, and DF. Three (7.5%) treatments were completely free of any local, regional, or DF. The outcomes for 7 (17.5%) treatments were unknown due to lack of follow-up imaging. All patients, who died, developed LF. For those patients with distant metastasis, six metastasized to the lungs only (60%); one metastasized to the tracheoesophageal groove (10%); one metastasized to the hilar lymph nodes (10%); one metastasized to the subcarinal lymph nodes (10%); and one metastasized to both the lung and the periesophageal lymph nodes (10%). One patient with small cell carcinoma in the head and neck did not develop local, regional, or DF after the SABR, but died from multiple myeloma. For the other two patients free of local, regional, or DF, one had adenoid cystic carcinoma in the parotid and is still alive and the other had T3 N1 M0 medullary carcinoma in the thyroid and is also alive.

**Figure 1:**
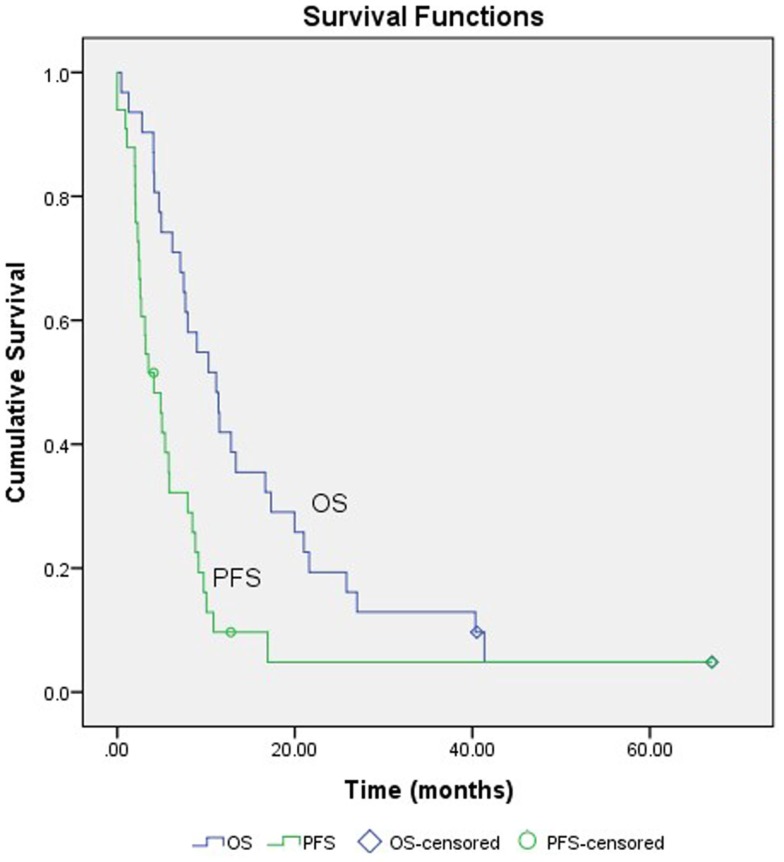
**Survival curves**. PFS, progression-free survival; OS, overall survival.

The median time-to-progression (TTP) was 3.3 months (range: 0–16.9 months). The estimated 3-month PFS, 6-month PFS, 9-month PFS were 55, 26, and 15%, respectively. Two patients (6.5%) were alive at the end of the follow-up period. For the 29 patients (93.5%) who died, the median time from the completion of first SABR to death was 10.3 months (range: 0.5–41.4 months). The estimated 1-year OS rate was 35%. The estimated 2-year OS rate was 12%. Both the PFS curve and the OS curve were shown in Figure 1.

### Dosimetric parameters

The median maximum radiation dose to the tumor was 51.3 Gy (range: 22.2–58.7 Gy). In addition, we measured the irradiated volume and the radiation dose to critical surrounding structures including the left and the right eye, left and right optic nerve, the chiasm, the brainstem, and the spinal cord. The detailed information was summarized in Table 2.

**Table 2:** **Dosimetric parameters for surrounding critical structures**.

Tissue	Volume (cm^3^), median (range)	Maximum radiation dose (Gy), median (range)
Left eye	9.1 (1.8 −12.6)	1.5 (0 −40.5)
Right eye	8.7 (5.7 −13.8)	3.05 (0 −28)
Left optic nerve	0.94 (0.4 −1.5)	9.9 (0.72 −47.5)
Right optic nerve	1.1 (0.4 −2)	7 (0.93 −48.5)
Chiasm	0.8 (0.3 −4.7)	5.5 (0.5 −43.4)
Brainstem	25 (6.6 −57.2)	14.7 (1.05 −39.9)
Spinal cord	25.9 (1.75 −62.7)	7.8 (0.97 −33.7)

### Toxicity assessment

Treatment was well-tolerated without any grade 4 or 5 treatment-related toxicities. All toxicities were listed in Table 3. Only 6 out of 40 (15%) SABR treatments led to significant toxicities (1 with acute grade 3 Erbitux associated rash, 1 with acute grade 3 alopecia, 1 with acute grade 3 dysgeusia, 1 with acute grade 3 hyperpigmentation1, 1 with late grade 3 headache, and 1 with late grade 3 trismus).

**Table 3:** **Toxicities after treatment**.

Adverse event	Acute (<90 days) (*n* = 29)	Late (>90 days) (*n* = 5)
**Erbitux™ associated rash**
Grade 1	2 (6.9%)	
Grade 2	2 (6.9%)	
Grade 3	1 (3.4%)	
**Nausea**		
Grade 2	2(6.9%)	
**Trismus**
Grade 3		1 (20%)
**Alopecia**		
Grade 3	1 (3.4%)	
**Pain**		
Grade 2		3 (60%)
**Dysphagia**		
Grade 1	1 (3.4%)	
Grade 2	1 (3.4%)	
**Xerostomia**		
Grade 1	2 (6.9%)	
Grade 2	1 (3.4%)	
**Mucositis**		
Grade 1	2 (6.9%)	
Grade 2	4 (13.8%)	
**Dysgeusia**		
Grade 1	2 (6.9%)	
Grade 3	1 (3.4%)	
**Telangiectasia**
Grade 1	1 (3.4%)	
**Skin atrophy**		
Grade 2	1 (3.4%)	
**Headache**		
Grade 2	1 (3.4%)	
Grade 3		1 (20%)
**Odynophagia**		
Grade 1	1 (3.4%)	
**Epistaxis**		
Grade 1	1 (3.4%)	
**Hyperpigmentation**		
Grade 3	1 (3.4%)	

## Discussion

Locally advanced or recurrent skull base malignancies have a very poor prognosis and are frequently inoperable due to the risk of severe brainstem and cranial nerve morbidities (15, 16). Re-irradiation of these patients is also clinically challenging due to the tumor’s proximity to critical neurovascular structures. Recently, fractionated stereotactic radiotherapy has become an attractive option to re-treat skull base malignancies, but there is no consensus on the optimal dose and fractionation for SABR as it applies to skull base malignancies. SABR is uniquely suitable for treating skull base malignancies as it is non-invasive and can target the tumor with great precision and conformity. However, there is very limited literature on the utilization of SABR for re-irradiating malignant SBT. To our knowledge, our study is the first to report toxicities of SABR for treating locally advanced or recurrent skull base malignancies with prior EBRT.

Cmelak et al. (17) reported a study of 47 patients with 59 malignant SBT. Among these patients, 37 were treated for 48 skull base metastases or local recurrences from primary head and neck cancers without previous irradiation. Eleven were treated for primary nasopharyngeal carcinoma using radiotherapy as a boost after a course of fractionated radiotherapy (64.8–70 Gy) without chemotherapy. The median tumor size was 8 cm^3^ (range: 0–51 cm^3^). A median radiation dose of 20 Gy (range: 7–35 Gy) was typically delivered in a single fraction. The median follow-up time was 9 months (range: 1–60 months). The crude local control rate (LC) was 33/48 (69%) during the follow-up period. Survival was not reported. Major complications developed in 5 out of 59 treatments, including three cranial nerve palsies, one CSF leak, and one trismus of unknown grade.

Miller et al. (18) reported a study of 32 patients with 35 newly diagnosed or recurrent malignant SBT treated with the Leksell Gamma unit. The median tumor size was 14.6 cm^3^ (range: 2.9–52.1 cm^3^). The median radiation dose was 15 Gy (range: 12–20 Gy) delivered in a single fraction. Three-year LC was 78% and 3-year OS rate was 72%. One patient received retreatment with hyperfractionated EBRT of 31.2 Gy about 1.7 years after the radiotherapy. Two patients with recurrent adenoid cystic carcinomas were previously treated with EBRT. One patient developed a radiation-induced optic neuropathy 12 months after radiotherapy. Majority of the patients had adenoid cystic carcinoma or chordoma.

Coppa et al. (5) reported a study of 31 patients with malignant SBTs. None of the patients were previously irradiated. The median follow-up time was 8.5 months. Ten (32%) patients were alive at the end of the follow-up period. The median OS was 8.6 months. For the 21 patients who died, the median time to death was 5.75 months. The median radiation dose was 25 Gy (range: 12.6–35 Gy) delivered in a median number of five fractions (range: 2–7). No significant toxicity was reported. The studies mentioned above were summarized in Table 4.

**Table 4:** **Previous experiences of skull base malignancies treated by stereotactic radiotherapy**.

Study	Median tumor size (cm^3^)	Techniques	No. of patients	Median f/u (months)	Median total dose (Gy)	Fractions	OS	LC
Cmelak et al.	8 (0 −51)	Stereotactic radiotherapy	47	9	20 (7–35)	1	N/A	69%
Miller et al.	14.6 (2.9 −52.1)	Gamma knife	32	27.6	15 (12–20)	1	3-year OS was 72%	78% at 3 years
Coppa et al.	18.3 (3.2 −206.5)	Cyberknife	31	8.5	25 (12.6–35)	5 (2–7)	5.75	74%
Our study	27 (2.4 −205)	Cyberknife, TrueBeam, Trilogy	31	11.4	44 (15–50)	5 (1–5)	10.3	3-month PFS was 55%

*f/u, follow-up; OS, overall survival; LC, local control*.

Though assessment of toxicity directly attributable to SABR was difficult as most patients underwent multiple surgeries, EBRT treatments, and chemotherapy sessions, no grade 4 or 5 acute or late radiation associated toxicities were noted in our study. There were five grade 3 toxicities. This included acute grade 3 rash, alopecia, and dysgeusia and late grade 3 trismus and headache. On the contrary, the single fraction radiotherapy studies reported relatively high rates of significant toxicities. We believe that the lack of significant toxicities is mostly due to delivering SABR in multiple fractions with high conformity and homogeneity. Fractionation and delivery of radiation every other day provide time for normal tissues to repair themselves between doses and therefore minimizes toxicities. Since our study is the first to report SABR for the re-irradiation of skull base malignancies, all the cited literatures were about using SABR for the treatment of locally advanced or recurrent skull base malignancies without prior irradiation. However, single fraction SABR caused significant late toxicities even in patients without prior irradiation, while multi-fraction SABR, like in our study, did not cause any grade 4 or 5 late toxicities in patients with prior EBRT. This shows that multi-fraction SABR helped to decrease the likelihood of late toxicities.

In addition, the high conformity of SABR ensures that irradiation to surrounding critical organs including left and right eye, left and right optic nerve, the optic chiasm, the brainstem, and the spinal cord was minimized as much as possible. In our study, the median maximum radiation dose to the left eye and the right eye was 1.5 and 3.05 Gy. The median maximum radiation dose to the left and right optic nerve was 9.9 and 7 Gy. The median maximum radiation dose to the optic chiasm was 5.5 Gy and the median maximum radiation dose to the brainstem was 14.7 Gy. The median maximum radiation dose to the spinal cord was 7.8 Gy. Shown by these dosimetric data, we can see that through its high conformity and precision, SABR minimized the radiation dose to surrounding critical organs while delivering a high dose to SBT. This makes SABR an attractive option for treating SBT becuase the biggest challenge is to avoid injuring its surrounding critical neurovascular structures.

Compared to previous studies, our study seems to have a relatively low control rate. However, it is worth noting that all the patients in our study have received previous EBRT. SABR was used for retreatment of inoperable locally advanced or recurrent skull base malignancies, not as a boost. Our median OS of 10.3 months was superior to the previously reported study regarding locally advanced or recurrent skull base malignancies as Coppa et al. only had a median survival of 5.75 months. In addition, our study had the largest tumor sizes among all the reported studies. The median tumor size in our study was 27 cm^3^ with a range of 2.4 to 205 cm^3^. Cmelak et al. had a median tumor size of 8 cm^3^; Miller et al had a median tumor size of 14.6 cm^3^; Coppa et al. had a median tumor size of 18.3 cm^3^. Furthermore, in our study, 17 patients (54.8%) had squamous cell carcinoma and only 6 (19.4%) had adenoid cystic carcinoma. Studies have shown that adenoid cystic carcinoma has a better prognosis than squamous cell carcinoma (19). In Miller et al., 12/32 (37.5%) patients had adenoid cystic carcinoma and only 8/32 (25%) had squamous cell carcinoma. 8/32 (25%) patients had chordoma, which is a rare, slow-growing malignant tumor.

Our dose and fractionation of 44 Gy in five fractions seem to be effective with acceptable long-term toxicities in this cohort of patients. However, this needs to be validated through prospective clinical trials. The dose ranges reported on Table 3 for critical organs were quite broad, and may not represent what is clinically appropriate. Currently, data are lacking regarding dose tolerances to these structures, especially in the setting of re-irradiation.

## Conclusion

Our study reported low toxicities with SABR for the re-irradiation of locally advanced or recurrent skull base malignancies, most likely due to the fractionation schedule and the high conformity of SABR, which ensures that irradiation doses to surrounding critical structures were minimized. Though fractionation seems to minimize toxicities, there is no consensus regarding the dose and fractionation of SABR for the treatment of skull base malignancies. Coppa et al. (5) reported a median radiation dose of 25 Gy delivered in five fractions and hypothesized that a higher average dose may still be associated with a low toxicity rate, which is supported by our study. In conclusion, SABR with a common regimen of 44 Gy delivered in five fractions has been shown to minimize toxicities in the treatment of locally advanced or recurrent skull base malignancies with prior EBRT at our institution.

## Conflict of Interest Statement

The authors declare that the research was conducted in the absence of any commercial or financial relationships that could be construed as a potential conflict of interest.
